# Deltas, freshwater discharge, and waves along the Young Sound, NE Greenland

**DOI:** 10.1007/s13280-016-0869-3

**Published:** 2017-01-23

**Authors:** Aart Kroon, Jakob Abermann, Mette Bendixen, Magnus Lund, Charlotte Sigsgaard, Kirstine Skov, Birger Ulf Hansen

**Affiliations:** 1 Department of Geosciences and Natural Resource Management, University of Copenhagen, Øster Voldgade 10, 1350 Copenhagen, Denmark; 2 Asiaq, Greenland Survey, Postbox 1003, 3900 Nuuk, Greenland; 3 Department of Bioscience, Arctic Research Centre, Aarhus University, Frederiksborgvej 399, 4000 Roskilde, Denmark

**Keywords:** Arctic catchments, Climatic drivers, Delta morphology, Delta regime, Fluvial discharges

## Abstract

A wide range of delta morphologies occurs along the fringes of the Young Sound in Northeast Greenland due to spatial heterogeneity of delta regimes. In general, the delta regime is related to catchment and basin characteristics (geology, topography, drainage pattern, sediment availability, and bathymetry), fluvial discharges and associated sediment load, and processes by waves and currents. Main factors steering the Arctic fluvial discharges into the Young Sound are the snow and ice melt and precipitation in the catchment, and extreme events like glacier lake outburst floods (GLOFs). Waves are subordinate and only rework fringes of the delta plain forming sandy bars if the exposure and fetch are optimal. Spatial gradients and variability in driving forces (snow and precipitation) and catchment characteristics (amount of glacier coverage, sediment characteristics) as well as the strong and local influence of GLOFs in a specific catchment impede a simple upscaling of sediment fluxes from individual catchments toward a total sediment flux into the Young Sound.

## Introduction

Ice, snow, and freezing temperatures are characteristic for Arctic coastal regions. The climate change in these areas induces an increase of air and seawater temperatures (Wadhams 2012), and the impact of climate change may be more pronounced for Arctic coastal environments (Lantuit et al. 2012). The sea ice is thinning, and its seasonal cover is decreasing (Barnhart et al. 2015). At the same time, the volume of the Greenland Ice Sheet is reducing, and this will lead to increasing freshwater fluxes toward the coastal zones and to a change in the local gravity field. A change of relative sea level over the next decades is still difficult to predict since the local balance between isostatic uplift and sea level rise is still unknown. On a decadal time scale, the response of the hydrological system to global warming is also measurable, especially in the Arctic. The freshwater fluxes from the melting glaciers will induce changes in Arctic Ocean dynamics. Overeem and Syvitski (2010) described the monthly freshwater fluxes of 19 large rivers between 1997 and 2007. They showed an increase in total annual water discharge (+10%), of melt month discharge (+66%), and a decrease in peak month discharge (−7%).

The seasonal variation in discharge is large: open waters and active rivers in summer and ice-covered coastal waters and inactive rivers in winter. Most of the traditional coastal processes by waves and tides are thus limited to summer and early fall. Many studies have focused on the seasonal discharge variation from the glaciers toward rivers, and only few have estimated the annual transport of suspended sediment between glaciers and coastal waters (Mernild and Hasholt 2009; Scott et al. 2014; Szpikowski et al. 2014). A few studies couple these annual sediment transport rates to sedimentation rates in fjords (e.g., Storms et al. 2012).

The seasonal variation of discharges in the Zackenberg River in Northeast Greenland has been studied by Rasch et al. (2000) and Søgaard et al. (2001). They used a limited amount of annual discharge curves and focused on the origin of water and sediment throughout the season. Hasholt et al. (2008) extended these studies by presenting longer time series and used exceedance curves of freshwater discharge to predict the return period of extreme events that were often observed in the river. A first attempt to upscale the discharge values from the Zackenberg River for the entire Young Sound was done on the basis of catchment sizes and not on the basis of discharge measurements (Mernild et al. 2007). They assumed that the glacier coverage and geology were homogeneous and used the catchment ratio to scale up the total freshwater discharge for the fjord (512 vs. 2620 km^2^). Their results indicated an increasing annual trend in river discharge. The freshwater discharge from the glacier to the fjord was also described with a hydrodynamic model (MIKE-SHE model; Mernild et al. 2008). The annual discharge could be reasonably estimated in some of the years. However, the contribution of the extreme events (GLOFs) is not possible to estimate in a hydrodynamic model.

The geomorphology of deltas is subdivided into a delta plain, a delta front, and a pro-delta (Anthony 2014). The delta plain is further divided into an upper area with mainly fluvial features and a lower area with tidal and estuarine features. The delta regime and associated delta morphology in the Young Sound area are dependent on the fluvial discharges and sediment load from the land, and on the waves and tides during the ice-free period from the fjord. These drivers are dependent on the catchment characteristics and the receiving basin characteristics, which can steer the sediment availability and the accommodation space.

The present study focuses on delta morphologies and delta regimes in the Young Sound area and discusses the spatial variability of the driving forces and its ability for upscaling.

## Field site description

### Physical setting of the Young Sound area

**Fig. 1:**
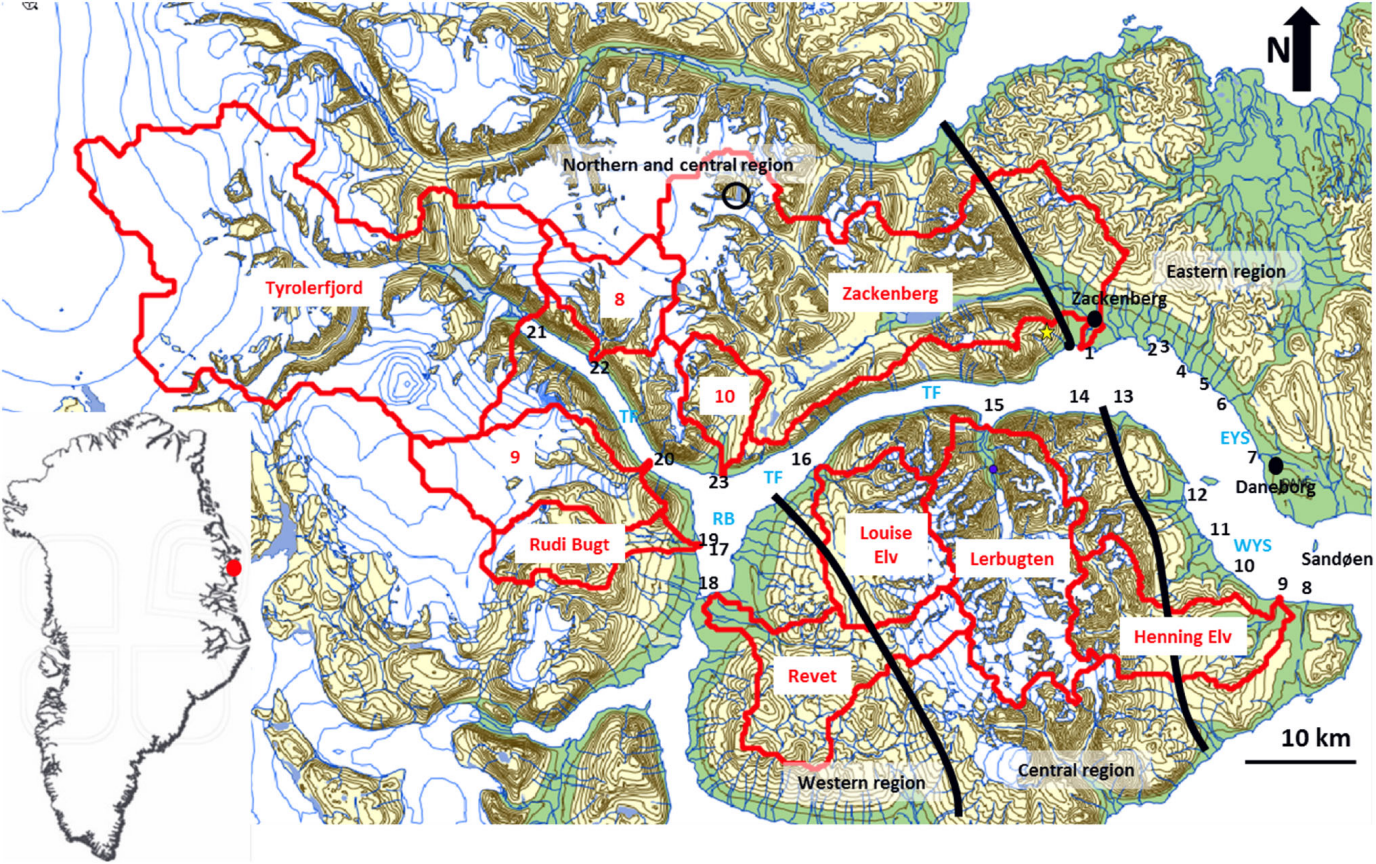
Map of Young Sound region in Greenland. Catchments (*red*), deltas (numbers in *black*), and boundaries between geological regions (*black lines*), glacier-dammed lake in the NV part of the Zackenberg drainage basin (*black open circle*), Zackenberg weather station and hydrometic station are located in the south-eastern part of the Zackenberg drainage basin within a km from each other (*black dot*). *EYS* Eastern shore of Young Sound, *WYS* Western shore of Young Sound, *TF* Tyroler Fjord, *RB* Rudi Bugt. Position of camera 1 (*yellow star*)

The Young Sound area (Fig. 1) is located in the High Arctic climate zone (Hansen et al. 2008) with continuous permafrost (Christiansen et al. 2008). Monthly mean air temperatures can reach below −20 °C with daily minimum below −30 °C in winter. Calm and weak winds from the north dominate in the winter months, but cyclone activity over the Greenland Ice Sheet or over the Greenland Sea regularly occurs and produces wind speeds >20 m s^−1^ and high temperatures. Mean monthly air temperatures are between 3.0 and 8.8 °C in high summer where air temperatures are >0 °C for most of the time. Air temperatures are <0 °C for most of the time from September to June. Most of the winds in summer are coming as a sea breeze from the south or southeast. The mean annual precipitation in Zackenberg is about 211 mm (1996–2013; Jensen et al. 2014) of which over 80% falls as snow. There is a large variability in local weather conditions due to complex topography and hence strong climate gradients.

The river catchments around the Young Sound/Tyrolerfjord in Northeast Greenland consist of glaciated and non-glaciated areas. The upper parts of some of the largest catchments are covered by glaciers (ca. 25% of the area covered), and meltwater is the major source of the freshwater discharge in summer. The altitude ranges between 0 and 1700 m above sea level (a.s.l.).

The Zackenberg River breaks up between 15 May and 12 June, and the Zackenberg River discharges exceed in ca. 10% of the time a value of 25 m^3^ s^−1^ (period 1996–2014). Extreme events during the summer months show discharges well over 200 m^3^ s^−1^ (e.g., Jensen et al. 2014; Søndergaard et al. 2015).

The coastal processes along the shores of the fjords and ocean are driven by tides and waves during the ice-free period, from mid-July until the end of September. The tide is mainly semidiurnal and has a clear variation over the neap-spring cycle. The fjord is a micro-tidal environment with a mean tidal range of ca. 0.9 m at Zackenberg and a spring-tidal range of ca. 1.7 m (Danish Maritime Safety Administration 2007). At the entrance of the fjord, the tidal ranges are similar (ca. 0.6 and 1.6 m, respectively) to M2, S2, K1 as dominant tidal constituents (Bendtsen et al. 2007). Almost all waves are locally generated, fetch limited, and low-energetic; only south-easterly winds have longer fetches and might produce waves over 2 m. Drifting ice in the fjord and along the shores might contribute to extra sediment transport activity along the shores in late spring, summer, and early fall.

### Geology and geomorphology of the Young Sound area

The Tyrolerfjord is the narrow innermost part of the fjord system in the west, and Young Sound is the wider outer part in the east toward open sea (Fig. 1). The fjord system is about 90 km long and 2–7 km wide and covers an area of 390 km^2^ (Bendtsen et al. 2007). The sill at the entrance of the Young Sound is only 45 m. The deepest part of the fjord system is located in Tyrolerfjord (maximum depth of 360 m). The mean depth is 80 m in the western Tyrolerfjord and Rudi Bugt, 183 m in the central and eastern Tyrolerfjord, and 91 m in the Young Sound (Bendtsen et al. 2007).

The study area is mountainous and cut by a number of valleys. The landscapes show a large variability in morphology and topography; it is situated in a transition zone between the glacial and ice-free terrestrial environments and the marine environments of the fjords and open waters (Fig. 1). The upper part is characterized by mountainous relief in bedrock, mainly granite, gneiss, and basalt (Koch and Haller 1971; Henriksen et al. 2009). The pro-glacial and fluvial valleys show alluvial fans, and these landscape features alternate with glacial and periglacial forms (Funder 1989; Christiansen and Humlum 1993; Hjort 1997; Christiansen et al. 2002). The lower parts of the areas show sedimentary coastal features like deltas, spits, salt marshes, and beach-ridge plains (Kroon et al. 2011; Pedersen et al. 2011).

The geology of the terrestrial part of the Young Sound area is divided into three regions: a western region, a central and northern region, and an eastern region (see Fig. 1, after Koch and Haller 1971). Rock formations and sediments in the western region are of carbonate origin with basaltic cores from Cretaceous–Tertiary eruptions, forming the top of the mountain ridges. The carbonate rocks of the Lower Cretaceous period are not very resistant against erosion and many small rivers deliver weathered sediments toward the coastal zone. The rocks in the central and northern region consist of the more resistant gneiss and granite formations of Cale-donian Crystalline complexes (Migmatite Gneiss, Mica-Schist and Biotite Gneiss, and Quartzite Schist to Gneiss; Koch and Haller 1971). The relief of this region is much more pronounced than that of its neighbors: mountains are higher and steeper and the upper parts of the mountains are covered by glaciers. Most of these glaciers drain into the larger valleys and the rivers discharge water and sediments toward several larger deltas at the end of glacial-shaped river valleys. The rock formations and sediments in the eastern region have again a Cretaceous origin with basaltic intrusions from Cretaceous–Tertiary eruptive. The mountains and valleys in the cretaceous rocks are less pronounced, but the basaltic intrusions are more resistant against erosion and form prominent cliffs and headlands in the coastal areas. Quaternary deposits, like moraines of the last glaciations, are observed in the coastal plains. The discharge and associated sediment fluxes are running from the glaciers to the fjord via pro-glacial and fluvial valleys towards the delta, and there are several sediment sources, like eroding river banks or alluvial fans, and sinks, like lakes, on their route. The substrate along the route is very variable, and the resistance of the material controls sediment availability, while the intensity and duration of the discharge controls sediment transportability.

## Materials and methods

### Climatic drivers, meteorology, sea-ice coverage, and waves

Meteorological data as air temperature and air pressure, wind velocity and wind direction, and snow depth were measured at a weather station in the Zackenberg valley (Fig. 1). The wind data were monitored with an interval of 10 min, the snow depth every three hours, and all other data were recorded hourly. The data described the general meteorological conditions from 1995 to 2014. The break-up and the freeze of the Young Sound were estimated from daily camera images from an automatic camera box ca. 490 m a.s.l. from 1999 to 2014. These data were used to compute the duration of the ice-free period.

The wind conditions were also used to estimate the potential impact of waves. The wind data of the ice-free period (here July, August, and September from 1996 to 2014) were split in 16 directions and 10 wind velocity classes. The fetch over the Young Sound was estimated for all directions at the Zackenberg shore (W-E orientation), and the angle between the shore-normal and the wind direction (*α*) was computed. Each mean wind velocity (v) of the 10 classes was converted into a wind-stress factor by applying the semi-empirical relation: wind-stress = 0.71 v^1.23^ (CERC 1984). The significant wave height (*H*_s_) was estimated using a nomogram with fetch and wind-stress as input variables (CERC 1984). Thereafter, *H*_s_ and *α* were used to compute the potential longshore sediment transport rate for sand at the breaker line (kg s^−1^ dry mass; CERC formula in Van Rijn 2002):

1 $$ Q_{\text{mass}} = 128 \, \left( {H_{\text{s}} } \right)^{2.5} \sin (2\alpha ). $$

The estimated *H*_s_ and *α* were assumed to be valid at the breaker line. The transport rate per wave height class and per direction was multiplied by the frequency of occurrence, using the wind statistics. Finally, the rates over all wave height classes were summarized, giving the potential longshore sediment transport rate for each direction.

The tidal range in the area was derived from the tide tables of Zackenberg (Danish Maritime Safety Administration 2007). These tide tables were assumed to be valid for the whole fjord area.

### Freshwater discharges and suspended sediment loads

The discharge (Q) has continuously been measured since 1995 in the lower end of the Zackenberg River, close to its mouth (see Mernild et al. 2008; Søndergaard et al. 2015) as part of the Greenland Ecosystem Monitoring program (GEM). Q was measured under different stages: vertical velocity profiles were measured with traditional instruments (propeller and Acoustic Current Doppler Profilers; ACDP) over a river cross-profile. Stage-discharge relations (Q-h relations) were set up for each year and were used to estimate the seasonal discharges. They have to be verified or renewed every year as the river bed topography is heavily impacted by reoccurring glacier lake outburst floods (GLOF). The discharges of three other rivers in the Young Sound area (Lerbugten, Rudi Bugt and Catchment 10) were measured during an intensive campaign in summer 2012. There, similar instrumentation was used, and local Q–h relations were derived. These measurements were running from 29 July until 19 August and were related to the Zackenberg River discharge curves (Larsen et al. 2012).

The extreme events of the river discharges were estimated with a simple peak-over-threshold approach using the annual discharge records with a measurement interval of 15 min. An event was defined as a period with discharges over a threshold value of 100 m^3^ s^−1^. The peak discharge of the event was recorded, and the duration of the event was defined as the period between the rapid rise and the rapid fall of the discharge curve.

The suspended sediment concentration (SSC) was based on filtered water samples from the Zackenberg River. These bottled water samples were taken twice a day (at 8 am and 8 pm) during most of the years. The sampling interval was more intense during extreme events (every 2–4 h) to better describe the peaks. The suspended sediment load was computed by multiplying the discharge by SSC.

### Geomorphology

The geomorphology was identified with the use of rectified and georeferenced aerial photos from 1973 and 1985 provided by the Danish Geodata Agency and satellite images from 2012. The areal sizes of catchments (km^2^) were estimated in ArcMap 10.3.1, together with the delta dimensions such as delta size and exposure. The delta sizes were defined by its length (straight distance of the delta stretch along the coast), protrusion, and shoreline delta length. These data were used to compute the ratios of length to protrusion and the curvature (shoreline delta length/length). The delta exposure definitions included the shore-normal angle of the delta stretch and the total amount of degrees with fetches over 3 km. This fetch angle could be composed of two sectors in cases where the shore-normal fetch was below 3 km. Fetches over 3 km were supposed to generate waves over 0.5 m. Successive georeferenced images were used to estimate changes in delta lobes, and shoreline erosion and accretion rates (m year^−1^) of the Zackenberg delta using the ArcGis based Digital Shoreline Analysis System (DSAS) provided by the U.S. Geological Survey (for detailed description see e.g., Thieler et al. 2009; Kabuth et al. 2014). Volumetric changes of deltas were estimated with these numbers.

## Results

### Temporal variation of climatic drivers, sea-ice content, and waves

The annual ice-free period near the Zackenberg delta showed large variability without a significant trend over the years (Table 1). The annual mean and standard deviation was 94 ± 16 ice-free days. The annual ice-free period was neither directly correlated to the length of the summer period with mean daily temperatures over 0 °C (*R* ^2^ < 0.10, not shown) nor with the day of ice break-up (*R* ^2^ < 0.10).

**Table 1:** Sea-ice statistics of the Young Sound based on daily images captured from an automatic camera box placed at 490 m a.s.l. on the Zackenberg mountain and from observations reported in the ZERO annual reports

Year	Ice break-up	Ice formation	Ice-free period
	Young Sound	Young Sound	Young Sound (days)
1996	13-07-1996		
1997	22-07-1997		
1998	22-07-1998		
1999	24-07-1999	30-09-1999	68
2000	08-07-2000	09-10-2000	93
2001	16-07-2001	21-10-2001	97
2002	08-07-2002	After 7/10, no images	
2003	08-07-2003	13-11-2003	128
2004	08-07-2004	01-11-2004	116
2005	07-07-2005	30-09-2005	85
2006	23-07-2006	06-10-2006	75
2007	17-07-2007	01-10-2007	76
2008	11-07-2008	18-11-2008	130
2009	14-07-2009	04-10-2009	82
2010	10-07-2010	12-10-2010	94
2011	11-07-2011	20-10-2011	101
2012	15-07-2012	10-10-2012	87
2013	02-07-2013	18-10-2013	108
2014	14-07-2014	28-10-2014	106

**Fig. 2:**
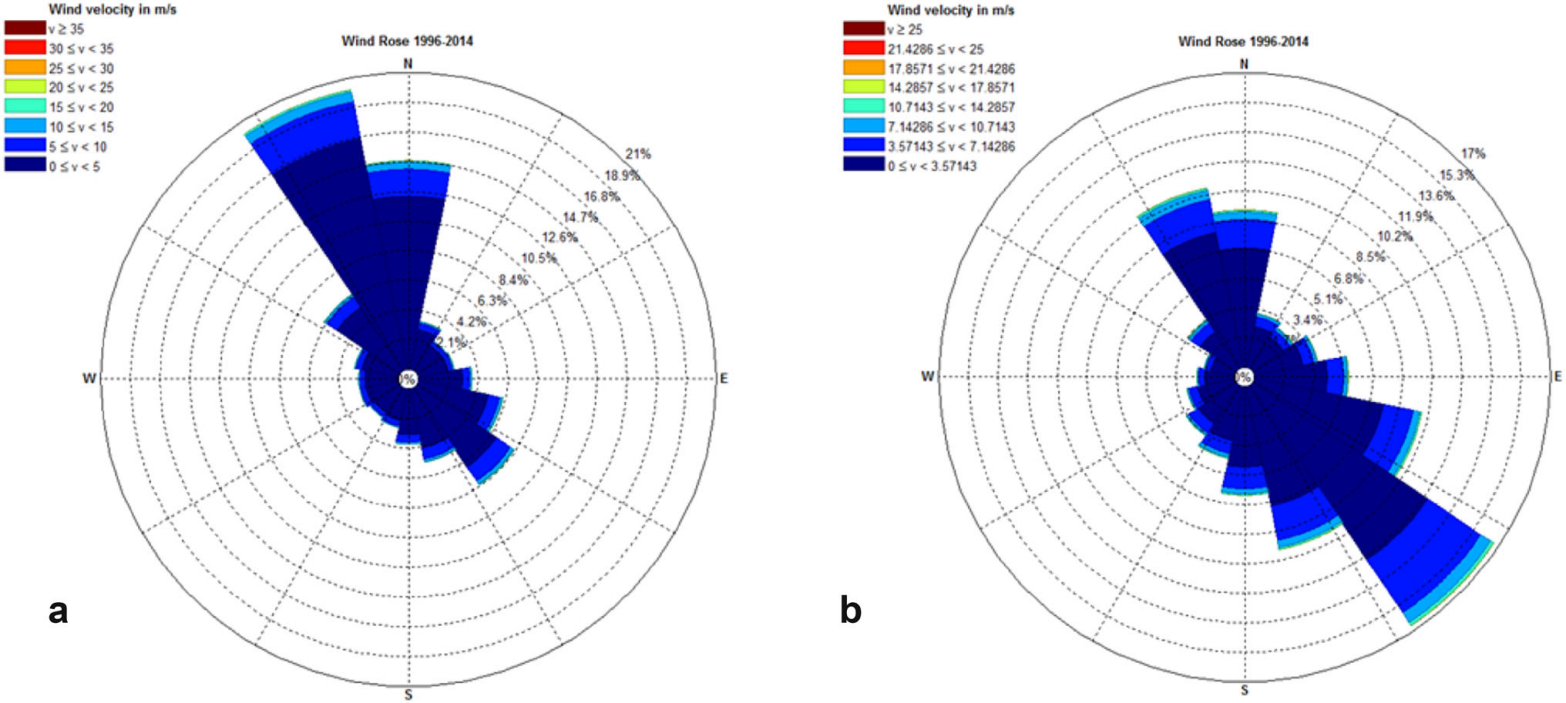
Wind roses of the Zackenberg Valley, based on 10 min data at 7.5 m over period 1996–2014. **a** All data, **b** July, August, and September

**Fig. 3:**
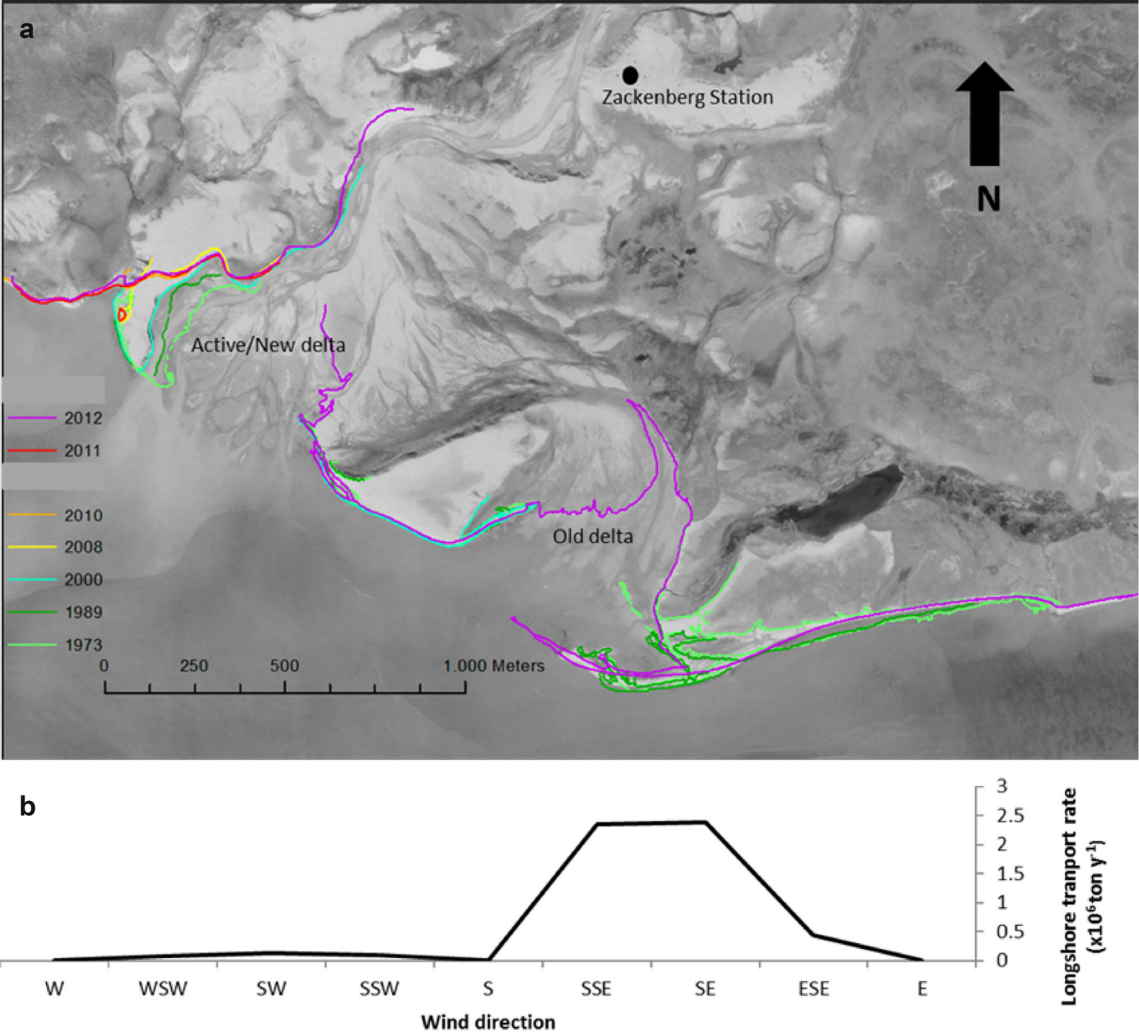
**a** Shoreline positions at high-tide in different years at the Zackenberg delta and **b** potential annual longshore sediment transport over the directions in front of the Zackenberg delta

The wind climate of the period 1996–2012 is presented in Fig. 2 for all months and for July–September, which is usually ice-free. The July-September wind climate was bidirectional with dominant wind directions from the SE and NNW. No storms were observed at Zackenberg in July–September. However, some years showed a gale or a strong gale in September, all out of NV to N directions. The winds blew over small fetches (<10 km) and could only generate moderate waves (*H*_s_). The potential annual longshore sediment transport rates by waves for the different wind directions in front of the Zackenberg delta are shown in Fig. 3. The waves from ESE–SSE contributed most to the potential transport from the east to the west, and this transport was much larger than the rates from the west to the east from SSW–W directions. The cross-shore sediment transport rates mimicked the wind climate in Fig. 2 and showed that the waves from SE-S will probably be most effective.

### Temporal variation of climatic drivers and fluvial discharges at Zackenberg

**Fig. 4:**
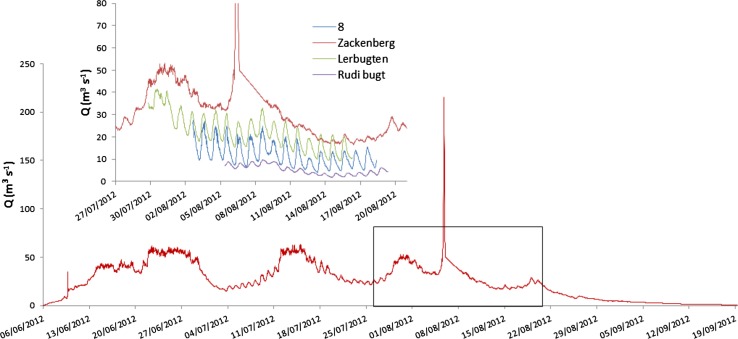
Discharge curves of Zackenberg River and three additional catchments over 2012. The panel with the discharge *curves* in late summer includes Catchment 8, Zackenberg, Lerbugten and Rudi Bugt (see locations in Fig. 1)

The mean annual discharges and associated suspended sediment loads of the Zackenberg River varied between 132 × 10^6^ and 338 × 10^6^ m^3^ year^−1^ and between 16.1 and 130 × 10^3^ t year^−1^ (Jensen et al. 2014). Both showed a large inter-annual variability with mean and standard deviations of 191 ± 47 × 10^6^ m^3^ year^−1^ and 39.1 ± 27.4 × 10^3^ t year^−1^, respectively, and did not show a significant linear trend over the years (both *R* ^2^ < 0.05, not shown). Melt water of glaciers was probably the substantial part of the total discharge, and annual discharge was not directly coupled to the precipitation (*R* ^2^ < 0.01, not shown). However, extreme events were regularly observed, and had a significant contribution to the annual values. The Zackenberg River discharge and suspended sediment curves showed a GLOF in August 2012 (Fig. 4). This GLOF originated at the AP Olsen glacier in the western part of the Zackenberg catchment (Fig. 1) and increased the suspended sediment concentration, and a tentative estimation of its impact on the annual load showed a contribution between 25 and 40%. All regular discharge records over the years 1996–2014 showed a total of 11 extreme events with discharges over 100 m^3^ s^−1^; at least two additional observed events occurred in Zackenberg River under winter conditions (November 26, 2008 and March 8, 2011). The origin of these events was manifold: rapid snow-melt, extreme rain events, GLOF, or a combination of these. The timing of the events differed over the years and directly produced large variability in erosional capacity. Occasionally occurring winter GLOFs were not assumed to cause suspended sediment load as water flows over a frozen snow-covered landscape. However, the break-up of ice cover and blocks of ice caused sediment transport on 26 November 2008.

### Spatial variation of fluvial discharges in the Young Sound area

The ten largest catchments and pronounced deltas in the Young Sound region are shown in Fig. 1. The morphometric description of catchments in the Young Sound area, the catchment size, and the ice-free area is given in Table 2. The Zackenberg catchment of 512 km^2^ was second in total size and first regarding its ice-free area.

**Table 2:** Catchment characteristics and discharge ratios (see Fig. 1 for location)

ID drainage basin	Area (km^2^)	Area ice-free (km^2^)	Dischargeratio Asiaq (%)	Discharge ratio area (%)	Discharge ratio area ice-free (%)	
1	513	421	100	100	100	Zackenberg
2	654	183		127	43	Tyrolerfjord
3	139	118		27	28	Revet
4	218	116	70	42	28	Lerbugten
5	125	114		24	27	Henning Elv
6	123	91		24	22	Louise Elv
7	73	60	20	14	14	Rudi Bugt
8	49	43		10	10	8
9	130	44		25	11	9
10	80	26	40	16	6	10

The Zackenberg River discharge in 2012 was coupled to discharge estimations of three other catchments (locations 15, 19 and 22 in Fig. 1). The amount of snow at the end of the winter 2012 was among the highest registered in Zackenberg, whereas the summer (June–August) was relatively dry with only 13 mm. The total annual discharge of Zackenberg River in 2012 was quite high (231 × 10^6^ m^3^ year^−1^). The Zackenberg River discharge curve and associated suspended sediment load of 2012 (Fig. 4) showed a seasonal trend with relatively high discharges and sediment loads just after the melt and relatively small sediment loads just before freezing set-in. The GLOF in 2012 was site-specific and absent in other discharges in the Young Sound area (Fig. 4). The annual estimates of discharges in three other catchments (Larsen et al. 2012) are presented in Table 2. These annual estimates were crude because they were based on measured water levels and discharge estimations over a three-week period of the ice-free season (see Table 2). However, all estimates of annual discharges were much smaller than those of Zackenberg River, but substantially larger than discharge ratio estimates based on catchment area as done by Mernild et al. (2007).

### Spatial variation of delta morphology

**Fig. 5:**
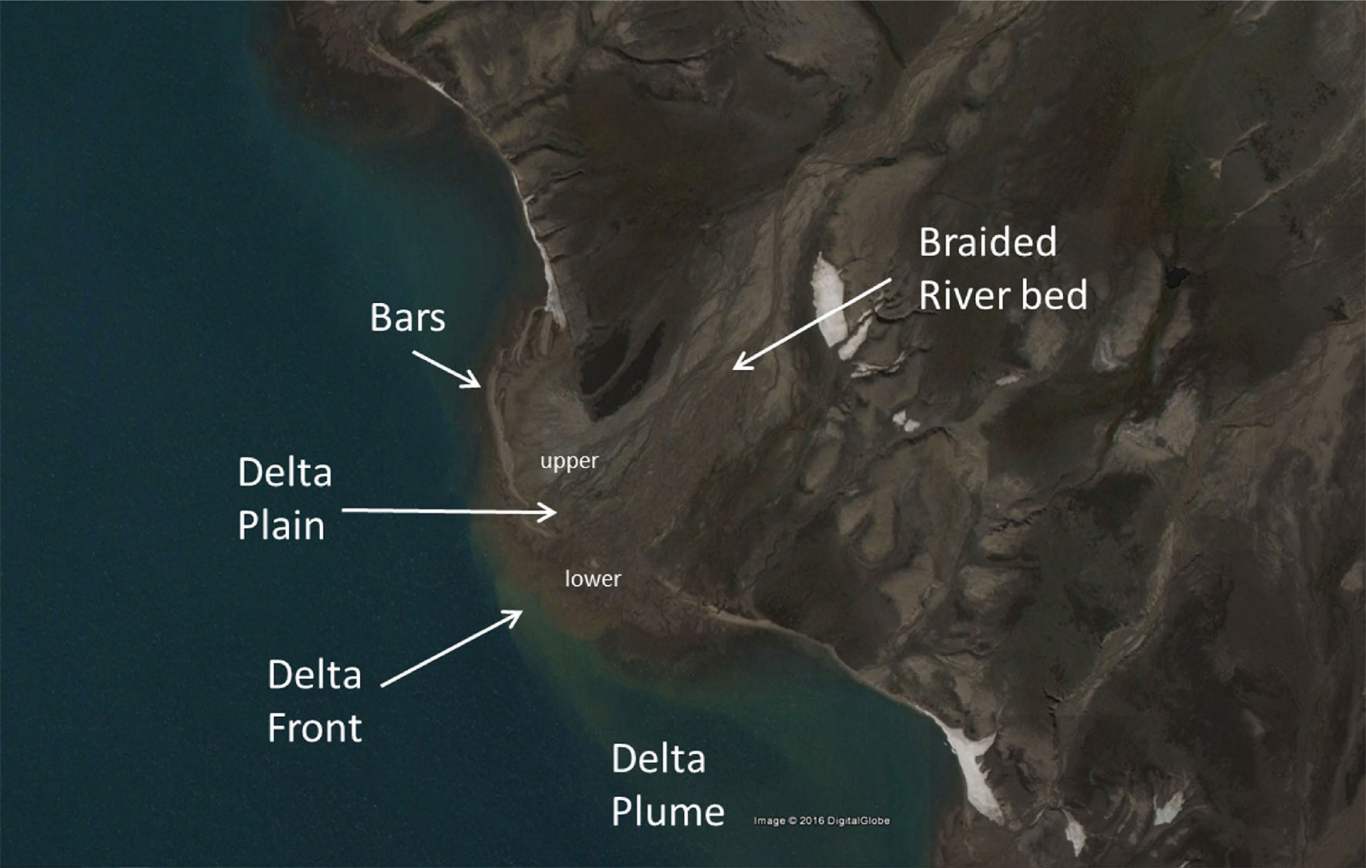
Delta terminology (delta 4 in Fig. 1)

The morphometric characteristics of 23 deltas in the Young Sound area are presented in Table 3. The deltas were subdivided into 4 regions with respect to their location: the eastern and western shore of Young Sound (EYS; WYS), the Tyrolerfjord, and Rudi Bugt (see Fig. 1 for locations). The catchments connected to deltas in EYS and WYS were mainly located in the geologic eastern region, those of Tyrolerfjord in the northern and central region and those of Rudi Bugt in the western region. The receiving fjord characteristics also differed: larger dimensions of the delta plain and delta front were associated with shallower fjord topography (see EYS deltas). The slopes of the central part of the Tyrolerfjord were steep and only coarse-grained fan deltas were observed (no delta plain). All other deltas had a delta plain, and coarse-grained deltas were of the braid-delta type. All the three largest deltas in the area (Zackenberg, Tyrolerfjord, and Lerbugten) showed a well-developed delta plain with a dominance of fluvial braided channels. The smaller deltas in the EYS and WYS also showed a distinct delta plain with an upper fluvial part divided from the lower delta plain by sandy bars (Fig. 5). These sand bodies were wave-generated berms or spits.

**Table 3:** Delta characteristics (see Fig. 1 for location)

Delta number	Fjord part	Geology	ID drainage basin	Delta size					Delta exposure				
				Length (m)	Width (m)	Shore length (m)	Curvature	Ratio Length/width	Shore-normal (°)	Fetch angle >3 km (°)	Normal Fetch (m)	Fringing bars	Multiple channels
1	E Young S	2	1	749			1.00		216	124	5800	Y	Y
2	E Young S	2		511	100	566	1.11	5.11	103	29	700	Y	
3	E Young S	2		283	101	377	1.33	2.80	261	87	680	Y	
4	E Young S	2		474	71	499	1.05	6.68	248	108	8700	Y	
5	E Young S	2		718	258	939	1.31	2.78	238	141	6250	Y	
6	E Young S	2		433	132	527	1.22	3.28	248	165	6100	Y	
7	E Young S	2		839	109	891	1.06	7.70	257	162	4000	Y	
8	W Young S	2		254	52	281	1.11	4.88	20	174	6100	N	
9	W Young S	2	5	1076	183	1157	1.08	5.88	30	164	6470	Y	
10	W Young S	2		1349	245	1484	1.10	5.51	66	179	6500	Y	
11	W Young S	2		1100	258	1237	1.12	4.26	100	82	7040	Y	
12	W Young S	2		264	77	331	1.25	3.43	46	164	6100	Y	
13	W Young S	2		1080	392	1396	1.29	2.76	24	310	6170	Y	
14	W Young S	1		878	196	952	1.08	4.48	18	163	4330	N	
15	Tyrolerfj	1	4	1182			1.00		28	71	3500	N	
16	Tyrolerfj	1	6	595	93	609	1.02	6.40	346	107	2450	N	
17	Rudy Bugt	3		1268	369	1438	1.13	3.44	308	95	2900	Y	
18	Rudy Bugt	3	3	5462	2545	7608	1.39	2.15	307	37	9880	Y	Y
19	Rudy Bugt	3	7	957	144	1025	1.07	6.65	88	186	4500	Y	
20	Tyrolerfj	1	9	574	332	945	1.65	1.73	56	55	1250	N	
21	Tyrolerfj	1	2	498–855			1.00		149	28	5300	N	Y
22	Tyrolerfj	1	10	1252	435	1533	1.22	2.88	235	58	1020	N	
23	Tyrolerfj	1	8	272	89	298	1.10	3.06	218	131	4400	N

**Fig. 6:**
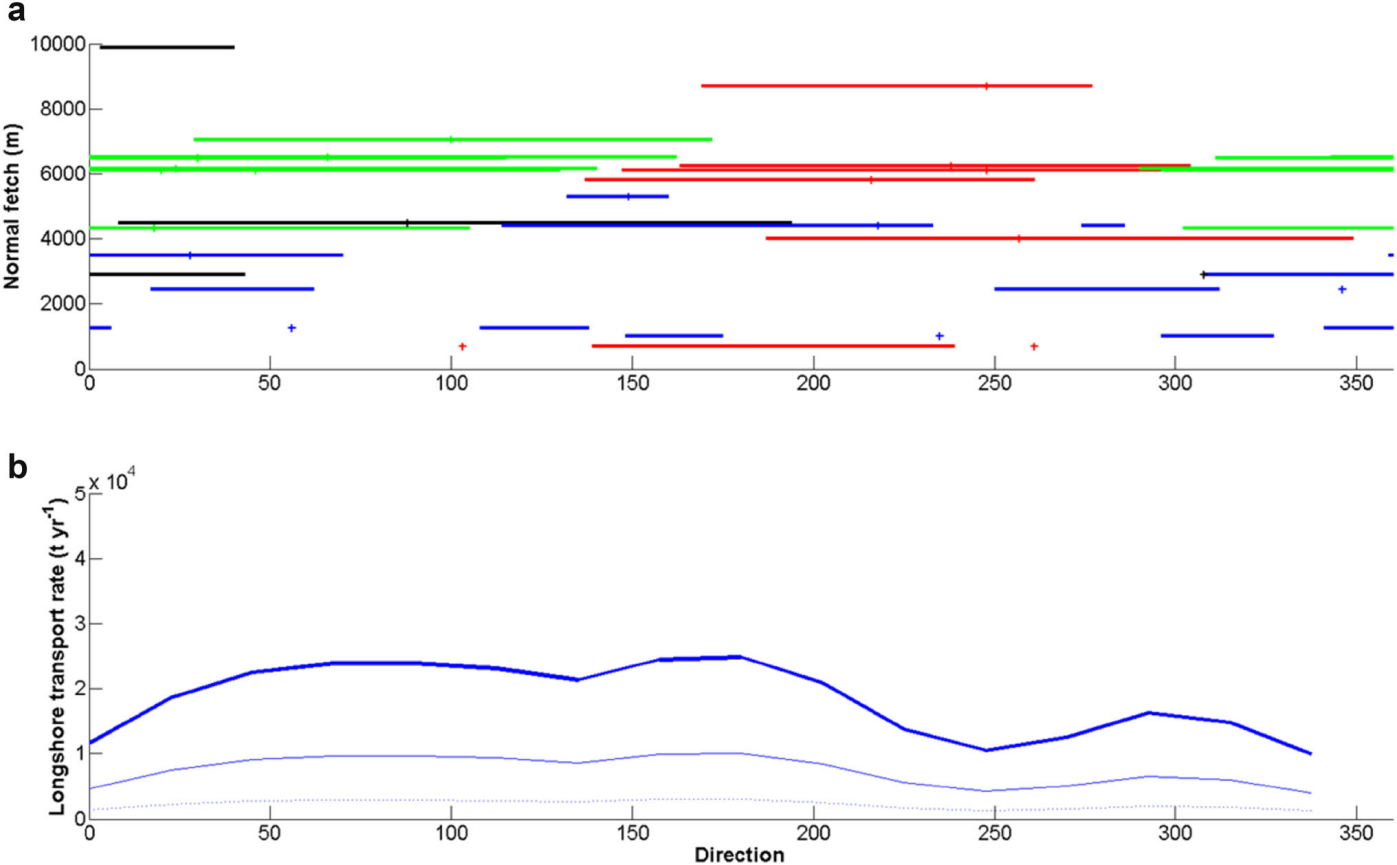
**a** The direction of fetches over 3 km at each delta in relation to the shore-normal fetch, and **b** the potential longshore sediment transport rates for the directions (*H*_s_ = 0.34 m (*dots*), 0.55 m (*thin line*) and 0.79 m (*thick line*)). The colors in **a** refer to exposure of western Young Sound deltas (*green*), eastern Young Sound deltas (*red*), Rudi Bugt deltas (*black*) and Tyrolerfjord deltas (*blue*)

The exposed deltas with the largest fetches were observed in EYS and WYS, while the Tyrolerfjorddeltas were more restricted with smaller fetches (Fig. 6a). Besides, many of these latter deltas had small open water angles (restricted by headlands or fjord topography) and the central normal fetch length was often below 3 km. All this indicated that only EYS and WYS, and to a lesser extent Rudi Bugt deltas were reworked by waves. The small deltas at the northern shore of central Tyrolerfjord did not have a delta plain and were thus of the coarse-grained fan delta type. These deltas were connected to small catchments and not to glaciers.

The wave impact on the deltas is further illustrated in Fig. 6b. The potential longshore transport over all directions was computed for a mean fetch of 3 km for three H_s_: 0.34, 0.55 and 0.79 m, representing conditions from low-energetic to moderate energetic. The frequency distribution of the associated wind velocities at Zackenberg was used and the pattern clearly showed a large increase of potential longshore sediment transport with increasing H_s_. The deltas in WYS were most sensitive to potential longshore transport and the maximum potential transport was close to the E-W shoreline orientation as in the Zackenberg case (Fig. 3). The potential cross-shore transport by waves mimicked the wind directions (Fig. 3a) with a clear dominance of waves on SE facing shores. The impact of waves was only estimated for local generated sea waves. These were assumed to be dominant in the whole area. However, swell waves from the open sea were sometimes observed at the mouth of Young Sound near the island ‘Sandøen.’

The three major deltas with a distinct multi-channel braided upper delta plain showed a mean shoreline accretion over the last decades. The mean shoreline change at the Zackenberg delta was in the order of 1 m year^−1^. However, there was a large variability in shoreline changes on the local scale along the delta (see Kroon et al. 2011 for more spatial and temporal details along the Zackenberg delta). The avulsion of a channel on the delta directly changed the erosion and accumulation patterns; the shoreline changes were often an order of magnitude larger than the mean shoreline changes for the whole delta.

## Discussion

Most deltas in the Arctic are of the Gilbert-type (Gilbert 1885). They are characterized by their steep delta front and tripartite structure and appear to be common in (a) tectonically active areas of high relief, (b) glacier marginal settings, and (c) in fjord settings (Corner et al. 1990). They develop well with an abundant supply of relatively coarse sediment (sand and gravel) in a relatively deep basin in a sheltered low-energy marine environment (see review in Corner et al. 1990). The deltas in the Young Sound area also fit in this delta type. However, the deltas of EYS and WYS do show significant reworking of the sandy sediments on the upper delta plain. This is probably caused by differences in the availability of sandy sediments in the distinct geological regions and by differences of potential reworking by waves. The Zackenberg delta for instance gets a lot of sediments from the eastern part of its catchment area and is optimally exposed to the dominant SE waves during July–September. This is directly reflected in the spit migration to the west and the onshore movement of the spit by cross-shore wave processes like swash and overwash (Fig. 3; Kroon et al. 2011). The EYS and WYS deltas show wave reworking and formation of swash bars fringing the upper delta plain. The micro-tidal environments in the fjord enhance the onshore migration of the swash bars fringing the upper delta plain (Masselink et al. 2006). The deltas in Tyrolerfjord are coupled to steep catchments of the central and northern region and to the deepest and narrowest part of the fjord system. They are very restricted by hard-rock topography and hardly exposed to waves. Delta plains are only developed in Tyrolerfjord when the sediment availability is large enough; otherwise, coarse-grained delta fans occur. Fringing swash bars on the delta plain are always absent. Volumetric changes of the delta front of all deltas in the Young Sound area are impossible to accurately estimate. Cross-shore profiles of the delta front and adjacent fjord bathymetry are not available for most of the deltas. Besides, the accommodation space is often large, due to the steep sides of the fjord, and fronts are very steep (slope of Zackenberg River delta front >6°).

The delta regime in Arctic deltas is related to fluvial discharges and sediment load out of the catchments and to the waves and tides during the ice-free open water period in the fjord. The discharges and sediment loads in the Zackenberg River are measured over 19 years, and there is a large variability on daily, seasonal, and annual time scales. The accuracy of annual suspended sediment load is low, because estimates are based on many assumptions (characteristic load for the whole cross-river profile); the total sediment load is still impossible to estimate (bedload problems). The unraveling of the drivers of the freshwater discharge is a multi-variate problem: snow coverage, precipitation, ice-melt, and insolation all do influence the daily and seasonal discharge curves. Besides, extreme freshwater discharge events do have a significant influence on the annual discharge rates and sediment loads, and some of these events are locally triggered like the GLOFs (Fig. 4). The sediment loads are not always directly related to the extreme events; heavy rainfall in August 1998 and September 2013 in the Zackenberg Valley triggered overland flow and contributed to a significant increase of sediment load under moderate freshwater discharges. Quantification of the waves in the area is indirectly done using the local wind climate and simple potential longshore transport equations. This could however successfully be used to explain the location and migration of swash bars and spits on the Zackenberg delta plain.

Simple estimations of freshwater discharges from the icecaps to the fjords from the Zackenberg catchment toward the whole Young Sound area are based on the dimensions of catchments (Mernild et al. 2007) or on comparison of discharge measurements in different catchments (this study). They all show large differences.

Spatial gradients in temperature, insolation, precipitation, cloud cover, and melt rates directly influence melt water discharge from the glaciers and therefore add a local signal to the individual catchment’s discharge. The daily cycle is visible in all discharge curves of the catchments (Fig. 4), but is more pronounced in those with larger glacier coverage (Table 2). However, the diurnal cycles are not always similar in areas with similar relative glacier coverage (Rudi Bugt and Zackenberg; Table 2 and Fig. 4). The cycle is far less visible in Zackenberg River, probably due to dampening of meltwater discharges by a large lake in its catchment. The modeled precipitation (data of the regional climate model HIRHAM for the period 1958–2012; Tedesco et al. 2016) in the area clearly shows a West–East gradient in the area (Table 4), with minimum annual values just east of the ice cap and maximum values near the shore. Spatial gradients in precipitation also results in differences of local freshwater supply by overland flow and subsurface (ground) water flow. Besides, the amount of glaciated area in the studied catchments varies in the area (Table 2) and non-glaciated catchments will probably not be impacted by GLOFs.

**Table 4:** Spatial distribution of annual precipitation in Young Sound region over the period 1958–2012. HIRHAM model runs (Tedesco et al. 2016)

Latitude	18	19	20	21	22	23	24	25
	East							West
Snow fall (mm)	274	259	252	254	251	241	178	196
Rain fall (mm)	59	36	32	27	16	9	9	18
Total (mm)	333	295	284	282	267	250	188	214

Spatial gradients in catchment characteristics also influence the suspended sediment loads towards the deltas. The geology of the three regions in the Young Sound area influence the sediment availability, sediment transportability, and the amount of vegetation on land and will indirectly influence the erodibility during for instance heavy rainfall events by overland flow. The western and eastern regions consist of less resistant hard rock that delivers abundant sediment. The fluvial discharges in these areas create deltas with extended delta plains of sand and gravel. These sand bodies are even reworked under low to moderate wave energies. Sand is probably less abundant in areas where hard rock is more resistant (central and northern region). Sand bodies are not observed in deltas related to these regions. This implies that the sediment transport capacity is not reached.

## Conclusions

There are over 20 deltas observed in the Young Sound fjord system. The largest deltas have a well-developed delta plain with multi-channels and are coupled to the largest catchments. The moderate deltas in the EYS and WYS have a clear delta plain and are coupled to catchments with relatively large sediment availability. These deltas are also located in the widest part of the fjord system and reworking of the delta plain by waves is optimal; swash bars along the fringes of the upper delta plain are commonly observed. The smaller deltas in the Tyrolerfjord are fluvial dominated and hardly exposed to waves. Swash bars are not observed on these deltas. They get relatively coarse sediment from quite resistant catchments, and the smallest deltas are fans and lack a delta plain.

Annual and seasonal patterns and extreme events of freshwater discharge are shown for the well-monitored Zackenberg River. The freshwater discharges clearly show a daily cycle with the highest levels at the end of the day in the summer season. Besides, extreme events like GLOFs are often observed (ca. 1 event per year) over the last decades. The simultaneously estimated freshwater discharges in other catchments within the Young Sound area show differences in patterns and rates. Daily cycles were always observed, but the extreme events often occur in a specific catchment. The differences in rates are due to spatial gradients in driving forces (snow and precipitation) and catchment characteristics (amount of glacier coverage, sediment characteristics) as well as the strong and local influence of GLOFs in a specific catchment hinders a simple upscaling of sediment fluxes from individual catchments toward a total sediment flux into Young Sound.
